# On-call work and depressive mood: A cross-sectional survey among rural migrant workers in China

**DOI:** 10.3389/fpsyg.2022.1068663

**Published:** 2023-01-09

**Authors:** Qingqing Xu, Liyun Wang, Yiwen Zhang, Xia Jiang

**Affiliations:** School of Economics, Qingdao University, Qingdao, China

**Keywords:** mental health, depressive mood, on-call work, gig economy, rural migrant workers

## Abstract

**Introduction:**

With the rapid development of China’s “gig economy,” the on-call work model has grown increasingly prevalent in China and has attracted a large number of rural migrant workers with its low employment threshold. However, this irregular employment mode may negatively impact the mental health of workers.

**Methods:**

This paper uses an ordinal logistic regression model to study the relationship between Chinese rural migrant workers’ on-call work and their depression.

**Results:**

The results showed that after controlling for relevant variables, the odds ratio of depressive mood among rural migrant workers engaged in on-call work was 1.22 (95% CI 1.04–1.43) compared with rural migrant workers who did not need to be on call. In further heterogeneity research, we found that on-call work is more likely to aggravate the depression risk of rural migrant workers who are highly dependent on the internet and have low-wage incomes.

**Discussion:**

This research suggests that appropriate measures should be taken to mitigate the negative impact of on-call work on the mental health of rural migrant workers, and more attention needs to be paid to the mental health of lower salaried and gig workers. This paper provides a valuable sample of Chinese rural migrant workers for theoretical research on the relationship between on-call work and mental health and confirms the relationship between the two. These results contribute new ideas to the theory and practice of psychological crisis intervention aimed at Chinese rural migrant workers.

## 1. Introduction

Mental health problems have become an increasingly prominent social issue, seriously affecting people’s lives and causing large economic losses to society (Allan et al., 2018). The severity of its harm makes it critical to identify relevant underlying drivers. Research has shown that a poor psychosocial work environment is associated with poorer mental health (Lee et al., 2020; Sprajcer et al., 2020). A large number of studies focus on the relationship between work schedules and workers’ mental health (Jiang et al., 2022). Zhao et al. (2019) pointed out that irregular shifts and long overtime are prone to triggering mental health problems in workers. At the same time, the impact of on-call work^1^ on employees’ physical and mental health has attracted academic attention. Scholars have found that on-call work makes employees more likely to suffer from sleep disorders (Jay et al., 2019), depression, anxiety, and other negative emotions, regardless of whether they are called in to work (Bamberg et al., 2012; Kovac et al., 2020). The reason for this is that when employees are called in, their working hours increase. Unpredictable needs or emergencies are the most common situations for on-call work (Gärtner et al., 2019); in these situations, mistakes can have serious consequences, and asking a colleague for help will disrupt the rest and entertainment time of the colleague and may also be interpreted as a sign of incompetence. Therefore, on-call work may make employees worry more and generate greater work and social pressure (Bamberg et al., 2012). Even if an employee is not called in, being on call means the possibility of being scheduled to work. This uncertainty makes it difficult for on-call employees to separate life from work, which is not conducive to work-life balance. Bamberg et al. (2012) point out that if work recovery is insufficient, the short-term effects of stress can develop into long-term effects, causing physical and mental health problems. Psychological alienation from work is an important aspect of job recovery, meaning the cognitive and emotional distance from work-related responsibilities and not thinking about work-related issues (Ziebertz et al., 2015). It is difficult for employees who are on call to maintain cognitive distance from their work, so physical and mental relaxation is limited, which may aggravate their anxiety and lead to fatigue (Ziebertz et al., 2015), sleep disorders (Vincent et al., 2021), low mood, depression, and other problems (Hall et al., 2017). In addition, some scholars have studied the impact of on-call work on employees’ daily lives. Golden (2015) argues that on-call work complicates the daily life of employees, adversely affects their social activities (Nicol and Botterill, 2004), and simultaneously affects their quality of life and that of their families (Baek et al., 2018; Karan et al., 2019), making it more likely to trigger family conflict (Golden, 2015). On-call work also requires workers to switch roles between life and work at any time and to be able to reach the workplace as soon as possible, thus constraining workers’ locations and leisure activities and limiting their social entertainment and fulfillment of family responsibilities (Woo, 2022).

Today, the on-call work model is no longer limited to *ad hoc* work arrangements but is becoming increasingly common. With the development of China’s digital economy, many changes have occurred in the labor market, among which the “gig economy” is an important new employment mode (Li and Yuan, 2019; Cai et al., 2021). China’s “gig economy” has the distinct characteristic of employing “temporary workers” (Muntaner, 2018). It is a new type of employment with relatively flexible work times and locations, mainly based on on-demand project-based work as the labor unit and project performance as the basis for remuneration (Wood et al., 2019). This work model is widely used in labor-intensive service fields such as express delivery, takeout, driving, and cleaning. It has attracted many individuals, including a large number of rural migrant workers from agriculture, due to its flexibility and low employment threshold (Bajwa et al., 2018). At the same time, with the rapid development of technologies such as the internet and artificial intelligence, traditional employment opportunities are gradually decreasing, and workers’ reliance on the “gig economy” is increasing. In this context, being on call has become a common mode of employment (Glavin and Schieman, 2022). However, being on call often means that workers’ mobile phones cannot be turned off for 24 h, and they must be ready to work at any time, which results in irregular work hours and easily leads to potential mental health problems (Hafeez et al., 2022).

Depression is the most common mental health problem among modern workers (Meunier et al., 2019) and is closely related to negative outcomes such as suicide (Vandivort and Locke, 1979; Martinengo et al., 2019); thus, when paying attention to the mental health of workers, depression is a problem that cannot be ignored. As a populous country, China has become one of the hardest hit areas for depression. According to a report released by the World Health Organization (2017), nearly half of the world’s individuals with depression lived in Southeast Asia and the Pacific region in 2015, with the number of depressed Chinese individuals at the forefront. In addition, the mental health problems of people with rural household registration (Zhang et al., 2022), low income, and low education are most prominent in China (Fu et al., 2021). Considering that the income level of rural migrant workers^2^ in China is limited (Yu et al., 2019) and the overall education level is low (Zhong et al., 2018), their risk of depression is higher than that of the general population (Ren et al., 2019). Therefore, this study selected Chinese rural migrant workers as research subjects to explore the relationship between on-call work and depressive mood. Data from the China Family Panel Studies (2020) showed that 35.38% of rural migrant workers scored more than 14 on the depression scale (CES-D8), while among urban workers, this proportion was only 30.78%. This finding indicates that the depressive tendency of rural migrant workers is greater than that of urban workers. Improving the psychological condition of rural migrant workers is therefore an urgent issue.

At present, there is a gap in research on the association between on-call work and the mental health of Chinese rural migrant workers. As an increasing number of rural migrant workers become gig workers, who are characterized by being on call, the potential mental health problems of these workers caused by being on call deserve more attention. Increasing their income is an important reason rural migrant workers engage in on-call work. A lower income reduces workers’ sense of social fulfillment and sense of control and security over their lives (Thomson et al., 2022), which tends to make them feel inferior and anxious. In contrast, if workers are able to earn a higher income, this helps improve their happiness and life satisfaction and alleviate the effects of negative factors, such as stress and loneliness, on their mental health (Zhu et al., 2020). Therefore, it is necessary to include income factors in the analytical model of mental health problems among on-call workers. It is also worth noting that the development of the internet is changing the way we work (Jacukowicz and Merecz-Kot, 2020; Mehta, 2020), and mobile internet technology has become an indispensable tool for gig workers (Vayre and Vonthron, 2019). On the other hand, the work of traditional on-call workers is less dependent on mobile internet. This makes the characteristics of on-call jobs performed by rural migrant workers vary with the level of internet use. Therefore, there may be differences in the effects of on-call work on depressive moods among rural migrant workers with levels of internet dependence. Exploring such differences is of great significance for identifying on-call workers at high risk of depression.

In summary, past research has shown associations between the irregular work pattern of being on call and mental health problems. This relationship has been confirmed in studies of specific occupational groups (e.g., doctors, midwives, firefighters, etc.) in Western culture countries, such as Finland, Canada, and the United Kingdom. However, little is known about whether the association still holds in China and in a wider range of occupational groups. Therefore, the first aim of this cross-sectional study was to examine the relationship between on-call work and mental health in a more general occupational group and to provide a valuable sample of Chinese rural migrant workers for relevant theoretical studies. Our second objective was to focus on the mental health problems of rural migrant workers in the context of the “gig economy” and to further examine the heterogeneity of the relationship between on-call work and depressive mood among rural migrant workers with different income levels and levels of internet dependence to provide a reference for developing psychological crisis intervention strategies for rural migrant workers.

## 2. Dataset

### 2.1. Sample selection

**Figure 1:**
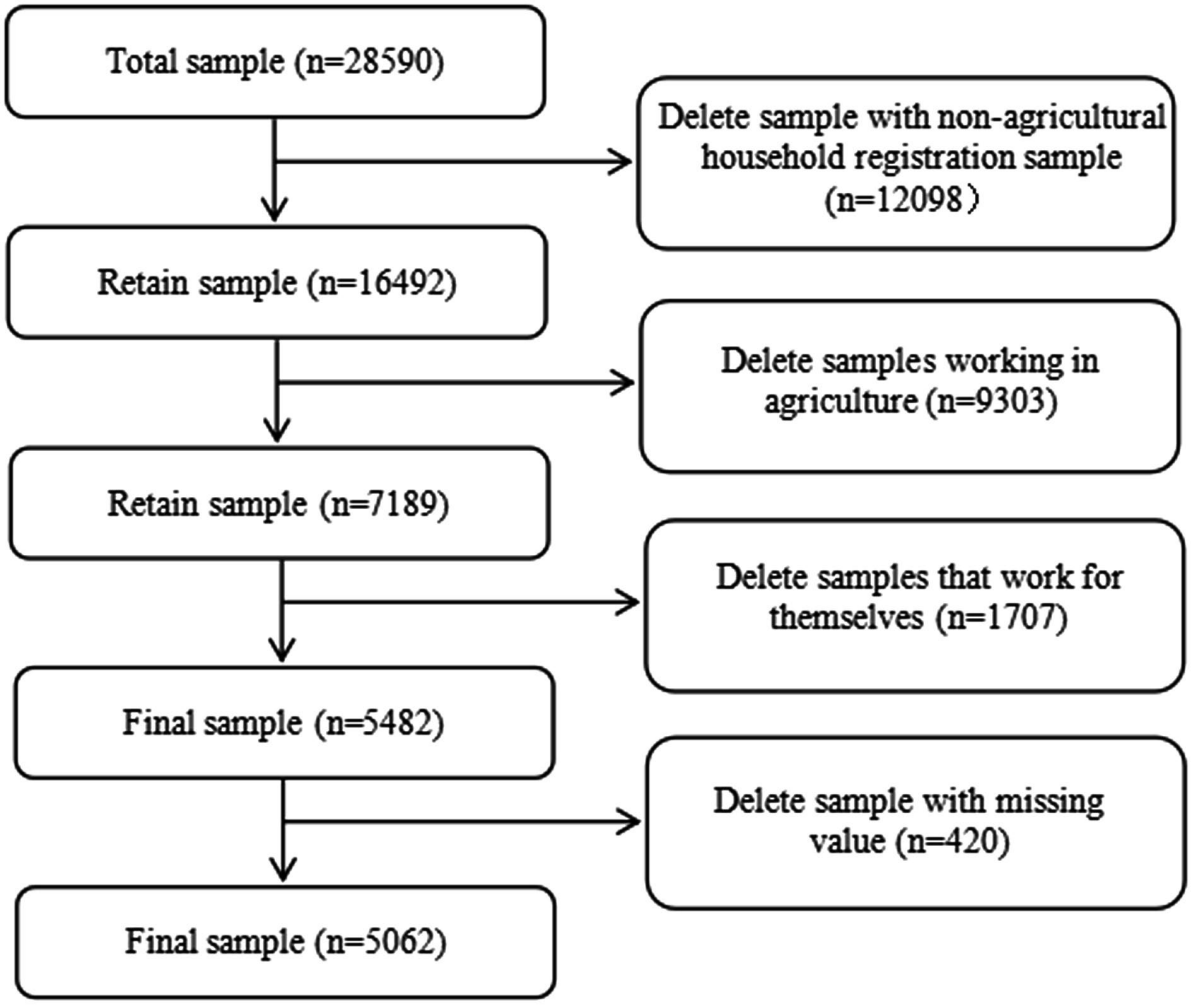
Research sample selection process.

The empirical data were from the 2020 China Family Panel Studies (CFPS) conducted by the Institute of Social Science Survey (ISSS) of Peking University. The survey was launched in July 2020 and continued until December 2020. Computer-assisted person-to-person interviews were used to extensively collect data at the individual, household, and community levels, reflecting societal, economic, demographic, educational, and health changes in China. It is a nationally representative social survey project in China (Xie and Hu, 2014). During sample screening, this study included the Chinese agricultural household registration population who were engaged in nonagricultural work as employees and earned a wage income (5482) as the research subjects and excluded individuals with unclear work characteristics (whether they needed to be on call) and mental health data (420). Thus, 5,062 valid subjects were included in the final analysis. The sample selection process is shown in Figure 1.

### 2.2. Measure

#### 2.2.1. Explained variable: Depressive mood

Depressive mood is assessed with eight items of the Center for Epidemiologic Studies Depression Scale (CES-D8), which is a simplified version of the CES-D20 (Radloff, 1977). Participants are asked how often certain feelings or behaviors occurred in the past week: (1) feeling down, (2) feeling strenuous to do anything, and experiencing (3) sleep disturbances, (4) pleasantness, (5) loneliness, (6) happiness, (7) sadness, and (8) unable to continue living. The above 8 questions were measured using a 4-point Likert scale (1 = less than a day, 2 = 1–2 days, 3 = 3–4 days, and 4 = 5–7 days). Two of the questions assessing positive emotions (pleasantness, happiness) are inversely scored. The final score represents the level of depressive mood and ranges from 8 to 32 points. Higher scores indicate higher levels of depression.

#### 2.2.2. Explanatory variable: On-call work

Whether the work requires being on call is measured by the question “Does your work require you to keep your mobile phone on for 24 h a day to be on call?” The answers were yes or no.

#### 2.2.3. Control variables

Many factors affect an individual’s depression. Drawing on the related study (Lin et al., 2020; Liu et al., 2021), the selection of control variables is based on five aspects (Table 1): individual characteristics, work characteristics, health behavioral characteristics, physical health, and socioeconomic status. Individual characteristics include age, gender, marital status (married, unmarried, divorced or bereaved), and education level (primary school and less, middle high school, high school/vocation school, junior college, college and more). Job characteristics include occupation category, whether a labor contract is signed (yes or no), and weekly working hours. Health behavioral characteristics are assessed by the following questions: “Have you smoked in the past month? “, “Have you drank more than three times a week in the past month? “, “Have you participated in physical activity in the past 12 months?” Two of the questions (smoking and drinking) measure unhealthy behaviors, and participation in physical activity reflects healthy behaviors. Answers are either yes or no. Physical health status is assessed by the following question: “How would you assess your health status?” (Bad, Fair, Good), and “Have you suffered from a chronic disease diagnosed by a doctor within the past six months?” (yes or no). Socioeconomic status is assessed by an individual’s assessment of income, with the question “How would you rate your income locally?” The question uses a 3-point Likert scale ranging from 1 (low) to 3 (high).

**Table 1:** Selection and measurement of control variables.

Primary indicators	Secondary indicators	Measurement	Question source
Individual characteristics	Age	Age	CFPS Individual Questionnaire A001
	Gender	Gender of interviewee	CFPS Individual Questionnaire QA002
	Marital status	Current marital status: married, unmarried, divorced or bereaved	CFPS Individual Questionnaire QEA0
	Education level	Highest completed (graduated) degree: primary school and less, middle high school, high school or vocation school, junior college, college and more	CFPS Individual Questionnaire W01
Work characteristics	occupation type	Industry of work	CFPS Individual Questionnaire QG302
	Labor contract	whether a labor contract is signed: 1 = Yes; 0 = No	CFPS Individual Questionnaire QG5
	Working hours	weekly working hours	CFPS Individual Questionnaire QG6
Health behavior characteristics	Smoking	Have you smoked in the past month? 1 = Yes; 0 = No	CFPS Individual Questionnaire QQ201
	Alcohol consumption	Have you drunk more than three times a week in the past month? 1 = Yes; 0 = No	CFPS Individual Questionnaire QQ301
	Physical exercise	Have you participated in physical activity in the past 12 months? 1 = Yes; 0 = No	CFPS Individual Questionnaire QP701
Health status	Self-rated health	How would you assess your health status? Bad, Fair, Good	CFPS Individual Questionnaire QP201
	Chronic disease	Have you suffered from a chronic disease diagnosed by a doctor within the past six months? 1 = Yes; 0 = No	CFPS Individual Questionnaire QP401
Socioeconomic status	Self-evaluation income	How would you rate your income at a local level? Low 1–2-3 High	CFPS Individual Questionnaire QN8011

## 3. Methodology

### 3.1. Descriptive statistics

Descriptive statistical analysis of variables is performed using the chi-square test or t test. In addition, in the CES-D20, with a score range of 0–60, Radloff (1977, 1991) proposed that a score of more than 16 points indicated depressive symptoms and that a score of more than 28 points indicated severe depression. According to this proportion, in this study, we consider rural migrant workers with a score of more than 14 on the CES-D8 to have depressive symptoms, and those with a score of more than 19 are considered to have severe depression. Based on this definition, this study analyzes the depressive tendencies in the sample.

### 3.2. Benchmark regression model

After adjusting for different potential confounding factors (individual characteristics, work characteristics, health behavior characteristics, physical health, socioeconomic status), this paper draws on relevant studies such as Cai et al. (2021) and Liu et al. (2021) using ordinal logistic regression models to estimate the relationship between on-call work and depressive mood and calculate odds ratios (ORs) and 95% confidence intervals (CIs). The ordered regression model is constructed as follows:


(1) $$P\left(y\leq j\right)=\frac{e^{\alpha_{j}-\overset{k}{\underset{k=1}{\sum }}\beta_{i}x_{ij}+\varepsilon_{i}}}{1+e^{\alpha_{j}-\overset{k}{\underset{k=1}{\sum }}\beta_{i}x_{ij}+\varepsilon_{i}}}$$

where j is the degree of depression; i is the subscript of the independent variable; P(y≤j) represents the probability that the degree of depression of rural migrant workers is less than or equal to j ; $X_{ij}$ is the independent variable; $\beta_{i}$ is the coefficient; $\alpha_{j}$ is the intercept, and $\varepsilon_{i}$ is the error.

To obtain formula (1), the logit processing algorithm adopted is shown in Appendix.

## 4. Results

### 4.1. Characteristics of the research subjects

The results of descriptive statistics (Table 2) show that the mean age (SD) of the rural migrant worker sample (*n* = 5,062) was 37.04 (11.82), and 2,493 (49.2%) of them had jobs requiring 24 h on call. The proportions of males and females in the total sample are 59.9 and 40.1%, respectively, of which 73.2% were married. The rural migrant workers averaged 55.63 (18.69) hours of work per week; 34% of the rural migrant workers had a habit of smoking, 14.4% drank alcohol at least three times a week, and 35.3% performed physical exercise. In addition, 52.9% reported that their economic status was at a moderate level. In descriptive statistics, the distribution of age, sex, marital status, working hours, smoking, drinking, physical exercise, and economic status is significantly different between the two groups with different job requirements (*p* < 0.05).

**Table 2:** Descriptive statistical characteristics of the samples.

Variables	On-call work		*p* value	Total, N (%)
	No, n (%)	Yes, n (%)		
Overall	2,569 (50.8)	2,493 (49.2)		5,062 (100)
Individual characteristics				
Age (year, mean ± SD)	35.71 ± 11.46	38.42 ± 12.02	<0.001	37.04 ± 11.82
Gender				
Female	1,206 (59.4)	823 (40.6)	<0.001	2029 (40.1)
Male	1,363 (44.9)	1,670 (55.1)		3,033 (59.9)
Marital status				
Unmarried	640 (56.4)	495 (43.6)	<0.001	1,135 (22.4)
Married	1822 (49.2)	1884 (50.8)		3,706 (73.2)
Divorce or bereaved	107 (48.4)	114 (51.6)		221 (4.4)
Education level				
Primary school and less	539 (50.0)	538 (50.0)	0.935	1,077 (21.3)
Middle school	947 (51.0)	911 (49.0)		1858 (37.6)
High school/vocation school	488 (50.2)	484 (49.8)		972 (19.2)
Junior college	342 (51.0)	329 (49.0)		671 (13.3)
College and more	253 (52.3)	231 (47.7)		484 (9.6)
Work characteristics				
Labor contract				
No	1,259 (51.4)	1,191 (48.6)	0.388	2,450 (48.4)
Yes	1,309 (50.2)	1,300 (49.8)		2,609 (51.5)
Working hours (mean ± SD)	54.59 ± 17.54	56.70 ± 19.75	<0.001	55.63 ± 18.69
Health behavior characteristics				
Smoking				
No	1842 (55.1)	1,500 (44.9)	<0.001	3,342 (66.0)
Yes	727 (42.3)	993 (57.7)		1720 (34.0)
Alcohol consumption				
No	2,270 (52.4)	2065 (47.6)	<0.001	4,335 (85.6)
Yes	299 (41.1)	428 (58.9)		727 (14.4)
Physical exercise				
No	1702 (52.0)	1,572 (48.0)	0.017	3,274 (64.7)
Yes	867 (48.5)	921 (51.5)		1788 (35.3)
Health status				
Self-rated health				
Bad	349 (49.9)	350 (50.1)	0.898	699 (13.8)
Fair	1,226 (50.9)	1,183 (49.1)		2,409 (47.6)
Good	993 (50.8)	960 (49.2)		1953 (38.6)
Chronic disease				
No	2,384 (51.1)	2,280 (48.9)	0.076	4,664 (92.1)
Yes	185 (46.5)	213 (53.5)		398 (7.9)
Socioeconomic status				
Self-evaluation income				
Low	793 (52.4)	720 (47.6)	<0.001	1,513 (29.9)
Fair	1,384 (51.7)	1,292 (48.3)		2,676 (52.9)
High	366 (43.4)	478 (56.6)		844 (16.7)

^*^Chi-square test and Student’s t-test were applied to detect distributed differences for categorical and continuous variables.

**Figure 2:**
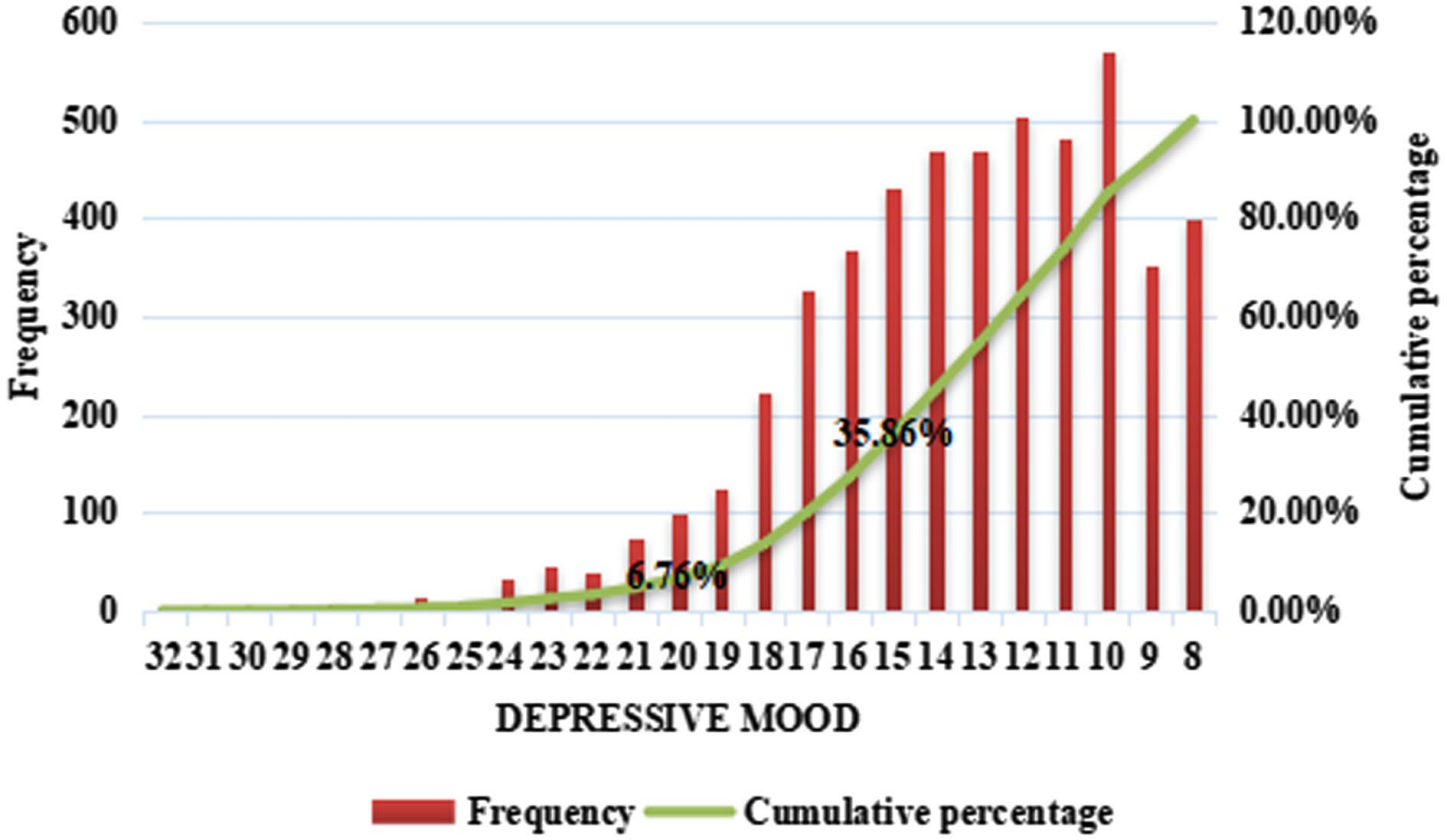
Frequency distribution and cumulative percentage of the degree of depression among rural migrant workers.

**Figure 3:**
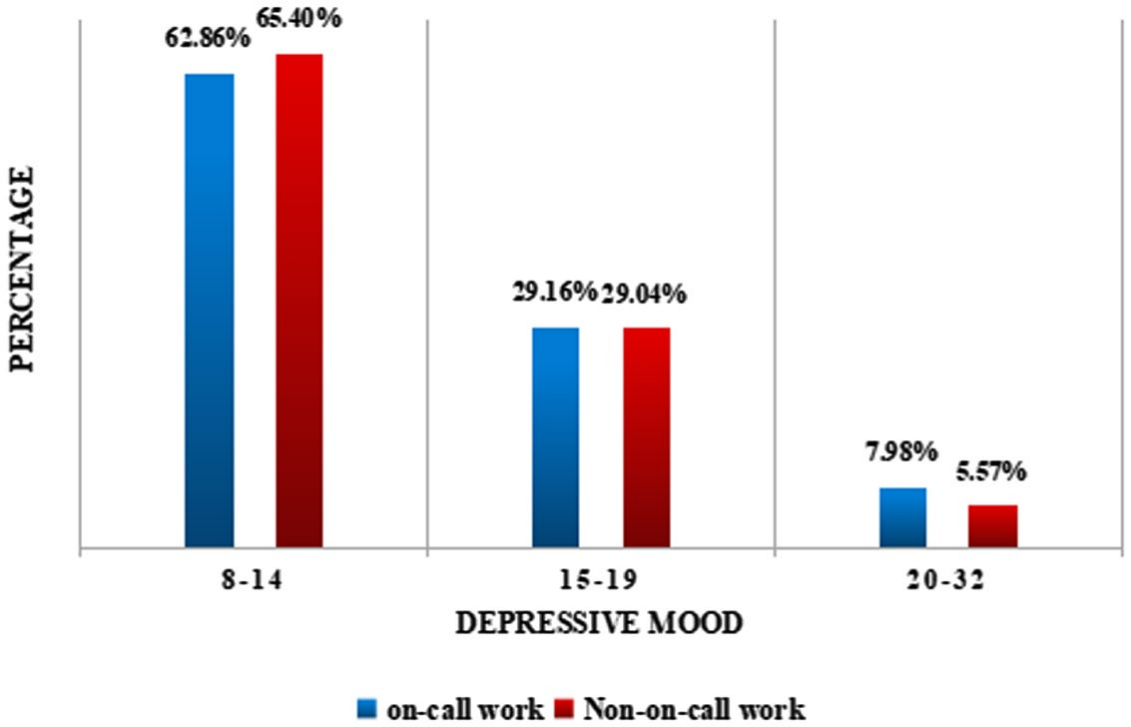
Comparison of depression scores between on-call and non-on-call rural migrant workers.

Figures 2, 3 reflect depressive tendencies among rural migrant workers. Figure 2 shows that 35.86% of the rural migrant workers in this study were depressed, and 6.76% of them had severe depression. Figure 3 further reveals that 37.14% (29.16% + 7.98%) of rural migrant workers who were on call were depressed, and 7.98% of them had severe depression. This proportion was higher than the depression rate of the sample of rural migrant workers who were not on call (34.61, 5.57%) and the depression rate of the total sample of rural migrant workers (35.86, 6.76%).

### 4.2. On-call work and depressive mood

Table 3 shows that, first, after controlling for occupational type factors, rural migrant workers who are engaged in on-call work are more likely to have mental health problems than those who do not need to be on call at all times (OR = 1.13, 95% CI 1.02–1.24, Model 0). Second, after adjusting for individual characteristics, on-call work significantly affects depression (*p* < 0.05). Finally, after adding work characteristics, health behavior characteristics, physical health, and socioeconomic factors to Model 1, the results show that on-call work is still an important cause of depression (Model 2). The tendency of on-call rural migrant workers to develop depression is 1.21 times higher than that of non-on-call rural migrant workers. In addition, the regression results of the control variables show that young married women are more likely to be depressed, and education level, working hours, health status, and economic status are also important factors affecting depression.

**Table 3:** Ordered logistic regression of on-call work and depressive mood.

Variables	Model 0		Model 1		Model 2	
	OR (95% CI)	*p* value	OR (95% CI)	*p* value	OR (95% CI)	*p* value
On-call work (ref: no)	1.13(1.02–1.24)	0.017	1.20(1.09–1.33)	<0.001	1.21(1.09–1.34)	<0.001
Age			0.99(0.98–0.99)	<0.001	0.98(0.97–0.98)	<0.001
Gender (ref: female)			0.81(0.73–0.90)	<0.001	0.77(0.68–0.87)	<0.001
Marital status (ref: unmarried)						
Married			0.82(0.71–0.94)	0.005	0.79(0.68–0.91)	0.001
Divorce or bereaved			1.68(1.26–2.22)	<0.001	1.53(1.14–2.04)	0.004
Education level (ref: Primary school and less)						
Middle school			0.74(0.64–0.86)	<0.001	0.74(0.64–0.86)	<0.001
High school/vocation school			0.66(0.56–0.79)	<0.001	0.69(0.58–0.82)	<0.001
Junior college			0.60(0.49–0.73)	<0.001	0.66(0.53–0.81)	<0.001
College and more			0.52(0.42–0.65)	<0.001	0.61(0.48–0.77)	<0.001
Labor contract (ref: no)					0.96(0.87–1.07)	0.481
Working hours					1.01(1.00–1.01)	<0.001
Smoking (ref: no)					1.24(1.09–1.42)	0.001
Alcohol consumption (ref: no)					1.01(0.86–1.17)	0.94
Physical exercise(ref: no)					0.91 (0.81–1.01)	0.081
Self-rated health (ref: bad)						
Fair					0.49(0.41–0.58)	<0.001
Good					0.26(0.22–0.31)	<0.001
Chronic disease (ref: no)					1.40(1.16–1.69)	<0.001
Self-evaluation income (ref: low)						
Fair					0.70(0.63–0.79)	<0.001
High					0.57(0.49–0.67)	<0.001
Occupation type	Control		Control		Control	
Observations	5,062		5,062		5,000	

*OR, odds ratio; CI, confidence interval; Coef., coefficient; S.E., robust standard error.

### 4.3. Robustness test

In this paper, two methods are used to further verify the reliability of the benchmark regression results. The first method is to modify the model (Model 3). Since the value category of the dependent variable depression level is 8–32, there are many values, and the interval is relatively uniform and can be approximated as a continuous variable (Wen et al., 2005), so this paper uses a multiple linear regression model to verify the robustness of the benchmark regression results. The second approach is to replace the assessment method of the socioeconomic status factor (Model 4). In the benchmark regression model, the socioeconomic status factor is measured by the individual’s self-evaluation of income, which is highly subjective and may cause cognitive bias. Therefore, the “wage income in the past 12 months” (take the logarithm) is used to evaluate the socioeconomic status of individual rural migrant workers. Table 4 shows the results of the two robustness tests. Overall, our econometric model is robust. The influence direction, significance, and odds ratio of the independent variables and control variables are basically consistent with the results of the benchmark regression model, indicating that the impact of on-call work on the depressive mood of rural migrant workers is relatively robust.

**Table 4:** Robustness test.

Variables	Model 3			Model 4		
	Coef.	S.E.	*p* value	OR	95% CI	*p* value
On-call work (ref: no)	0.398	0.105	<0.001	1.203	1.09–1.33	<0.001
Age	−0.039	0.006	<0.001	0.977	0.97–0.98	<0.001
Gender (ref: female)	−0.418	0.134	0.002	0.776	0.68–0.88	<0.001
Marital status (ref: unmarried)						
Married	−0.541	0.149	<0.001	0.787	0.68–0.91	0.001
Divorce or bereaved	0.875	0.322	0.007	1.584	1.19–2.11	0.002
Education level (ref: Primary school and less)						
Middle school	−0.603	0.151	<0.001	0.751	0.65–0.87	<0.001
High school/vocation school	−0.767	0.182	<0.001	0.698	0.59–0.83	<0.001
Junior college	−0.787	0.223	<0.001	0.662	0.54–0.82	<0.001
College and more	−1.035	0.243	<0.001	0.605	0.48–0.76	<0.001
Labor contract (ref: no)	−0.084	0.109	0.445	0.961	0.86–1.07	0.458
Working hours	0.013	0.003	<0.001	1.006	1.00–1.01	<0.001
Smoking (ref: no)	0.446	0.134	0.001	1.224	1.08–1.39	0.002
Alcohol consumption (ref: no)							−0.004	0.158	0.979	1.003	0.86–1.17	0.972
Physical exercise (ref: no)	−0.23	0.113	0.042	0.916	0.82–1.02	0.115
Self-rated health (ref: bad)						
Fair	−1.677	0.189	<0.001	0.475	0.40–0.56	<0.001
Good	−2.93	0.192	<0.001	0.239	0.20–0.28	<0.001
Chronic disease (ref: no)	0.6	0.209	0.004	1.431	1.18–1.73	<0.001
Wages income				0.968	0.94–1.00	0.031
Self-evaluation income (ref: low)						
Fair	−0.839	0.124	<0.001			
High	−1.177	0.161	<0.001			
Constant	17.321	0.415	<0.001			
Occupation type	Control			Control		
Observations	5,000			5,029		

*OR, odds ratio; CI, confidence interval; Coef., coefficient; S.E., robust standard error.

### 4.4. Heterogeneity analysis

Before the heterogeneity analysis, this paper examined the interaction effects of mobile device time online, wages and the on-call work mode on depression. Based on the results of the interaction effect analysis model, rural migrant workers are divided into three groups according to the tertiles of the daily mobile internet time, namely, a long internet time, medium internet time, and short internet time. Similarly, rural migrant workers are divided into three groups according to their wage income tertiles, namely, low wages, medium wages, and high wages, and then the heterogeneity of the relationship between on-call work and depressive mood is analyzed with different levels of internet dependence and wages.

#### 4.4.1. Interaction effect model

Table 5 shows that the interaction between the two factors of mobile device time online and wages and the on-call work mode is significant (*p* < 0.05). This indicates that the effect of on-call work on depressive moods among rural migrant workers varies with levels of internet use and wages. The development and popularization of the internet are closely related to the popularity of the on-call work mode. The difference in the degree of dependence of rural migrant workers on the internet also reflects the different characteristics of their on-call work. Gig workers are an important part of the on-call workforce. They often need to use the internet and mobile technology to receive work assignments and provide services on demand (Graham and Anwar, 2019), so they are highly dependent on the internet. In contrast, traditional on-call work is less reliant on the internet. Among rural migrant workers with different degrees of internet dependence, the difference in the impact of on-call work on depression also reflects the difference in the impact of traditional on-call work and new on-call work on depression. In addition, as a direct return for work, wages affect workers’ job satisfaction (Stander et al., 2019) and negatively regulate the effect of on-call work on depression. That is, higher wages alleviate the negative impact of on-call work on the mood of rural migrant workers.

**Table 5:** Analysis of interaction effects.

Variables	(a)			(b)		
	OR	95% CI	*p* value	OR	95% CI	*p* value
On-call work (ref: no)	1.2	1.08–1.33	0.001	2.29	1.26–4.17	0.006
On-call work*Wages				0.94	0.89–0.99	0.029
On-call work*Online time	1.03	1.01–1.05	0.014			
Age	0.98	0.97–0.99	<0.001	0.98	0.97–0.98	<0.001
Gender (ref: female)	0.78	0.68–0.89	<0.001	0.77	0.68–0.88	<0.001
Marital status (ref: unmarried)						
Married	0.79	0.68–0.91	0.001	0.78	0.68–0.90	0.001
Divorce or bereaved	1.46	1.09–1.95	0.01	1.58	1.19–2.11	0.002
Education level (ref: Primary school and less)						
Middle school	0.74	0.64–0.85	<0.001	0.75	0.65–0.87	<0.001
High school/vocation school	0.68	0.57–0.81	<0.001	0.7	0.58–0.83	<0.001
Junior college	0.65	0.52–0.80	<0.001	0.66	0.53–0.82	<0.001
College and more	0.59	0.47–0.75	<0.001	0.61	0.48–0.77	<0.001
Labor contract (ref: no)	0.96	0.86–1.07	0.459	0.96	0.86–1.06	0.41
Working hours							1.01	1.00–1.01	<0.001	1.01	1.00–1.01	<0.001
Smoking (ref: no)	1.24	1.09–1.41	0.001	1.23	1.08–1.40	0.002
Alcohol consumption (ref: no)	1	0.86–1.16	0.972	1	0.86–1.17	0.969
Physical exercise (ref: no)	0.9	0.81–1.01	0.072	0.92	0.82–1.02	0.126
Self-rated health (ref: bad)						
Fair	0.49	0.41–0.58	<0.001	0.48	0.40–0.56	<0.001
Good	0.26	0.22–0.31	<0.001	0.24	0.20–0.28	<0.001
Chronic disease (ref: no)	1.41	1.17–1.70	<0.001	1.43	1.19–1.73	<0.001
Self-evaluation income (ref: low)						
Fair	0.71	0.63–0.79	<0.001			
High	0.58	0.50–0.68	<0.001			
Occupation type	Control			Control		
Observations	4,975			5,029		

#### 4.4.2. Grouped by mobile internet time

Based on the analysis of the interaction effects, this study further examined the relationship between on-call work and depression in three groups with different degrees of internet dependence. Table 6 shows the results of the heterogeneity analysis. The findings demonstrate that at the 5% significance level, on-call work had a significant impact on depression among rural migrant workers who spent a long time on mobile internet. However, in the sample group of rural migrant workers with short and medium time online, the impact of on-call work on depression is not obvious. At the 10% significance level, on-call work had a significant impact on depression among the three groups of rural migrant workers, and on-call work was more likely to increase the risk of depression among rural migrant workers with long mobile internet time (OR = 1.22,95% CI 1.04–1.44). Although on-call workers who spend much time on the internet are not always gig workers, gig workers who are more reliant on the internet must have relatively long periods of time online because of the rigid demands of their work. Moreover, rural migrant workers make up a significant proportion of gig workers, so it can be inferred that there are many gig workers among on-call workers whose jobs require staying in close contact with the internet. On-call work is more likely to aggravate the depression among rural migrant workers with long mobile internet time, which indicates that the new gig labor is likely to put rural migrant workers at a higher risk of depression.

**Table 6:** Associations between on-call work and depressive mood, grouped by internet usage.

Variables	Short internet time			Medium internet time			Long internet time		
	OR	95% CI	*p* value	OR	95% CI	*p* value	OR	95% CI	*p* value
On-call work (ref: no)	1.2	0.98–1.47	0.072	1.16	0.98–1.38	0.093	1.22	1.04–1.44	0.017
Age										0.98	0.97–0.99	<0.001	0.99	0.98–1.00	0.049	0.99	0.97–1.00	0.081
Gender (ref: female)	0.84	0.65–1.09	0.195	0.75	0.61–0.92	0.007	0.76	0.61–0.94	0.013
Marital status (ref: unmarried)									
Married	0.61	0.41–0.91	0.016	0.83	0.63–1.10	0.196	0.78	0.64–0.96	0.018
Divorce or bereaved	1.13	0.59–2.14	0.714	1.46	0.86–2.48	0.156	1.43	0.92–2.22	0.114
Education level (ref: Primary school and less)									
Middle school	0.74	0.59–0.92	0.006	0.87	0.68–1.11	0.256	0.55	0.39–0.78	0.001
High school/vocation school	0.72	0.52–1.01	0.06	0.8	0.60–1.08	0.145	0.51	0.35–0.73	<0.001
Junior college	0.92	0.56–1.52	0.751	0.61	0.41–0.92	0.017	0.49	0.33–0.72	<0.001
College and more	0.63	0.29–1.39	0.253	0.8	0.53–1.20	0.279	0.42	0.28–0.62	<0.001
Labor contract (ref: no)	1.01	0.82–1.24	0.921	0.93	0.78–1.11	0.437	0.95	0.80–1.14	0.589
Working hours										1	1.00–1.01	0.172	1.01	1.01–1.02	<0.001	1.01	1.00–1.01	0.009
Smoking (ref: no)	1.02	0.79–1.31	0.902	1.2	0.97–1.50	0.099	1.45	1.16–1.80	0.001
Alcohol consumption (ref: no)	0.82	0.63–1.07	0.15	0.94	0.73–1.22	0.652	1.27	0.97–1.66	0.078
Physical exercise (ref: no)										0.69	0.54–0.89	0.004	1.04	0.86–1.26	0.668	0.94	0.79–1.11	0.447
Self-rated health (ref: bad)									
Fair	0.57	0.43–0.76	<0.001	0.33	0.25–0.45	<0.001	0.62	0.46–0.84	0.002
Good	0.29	0.21–0.38	<0.001	0.21	0.15–0.28	<0.001	0.3	0.22–0.41	<0.001
Chronic disease (ref: no)	1.37	0.99–1.90	0.058	1.35	0.98–1.85	0.067	1.5	1.08–2.08	0.015
Self-evaluation income (ref: low)									
Fair	0.63	0.50–0.80	<0.001	0.74	0.61–0.91	0.004	0.68	0.56–0.81	<0.001
High	0.6	0.45–0.78	<0.001	0.55	0.41–0.72	<0.001	0.57	0.43–0.74	<0.001
Occupation type	Control			Control			Control		
Observations	1,335			1707			1933		

#### 4.4.3. Grouped by wages

Similarly, based on the analysis results of interaction effects, this study further examines the relationship between on-call work and depression in three groups with different wages. The results (Table 7) show that on-call work has a significant effect (*p* < 0.05) on the depressive mood of rural migrant workers with low and medium wages but no significant effect on those with high wages. Income is the labor feedback of rural migrant workers engaged in on-call work. When wages are high, workers’ satisfaction increases and compensates for the negative impact of on-call work to a certain extent (Danish and Usman, 2010; Kronenberg et al., 2015; Qiu et al., 2021). In addition, according to the job demand-control model (Karasek, 1979), high job demands and low job control lead to high job stress. Workers’ income is closely related to their education level and workability (Griliches and Mason, 1972), and workers with lower incomes have lower levels of education and workability. They are more likely to be in environments with high work demands and low work control, to face greater hindrance stress and to develop psychological problems.

**Table 7:** Associations between on-call work and depressive mood, grouped by income level.

Variables	Low wages			Medium wages			High wages		
	OR	95% CI	*p* value	OR	95% CI	*p* value	OR	95% CI	*p* value
On-call work (ref: no)	1.37	1.15–1.64	0.001	1.22	1.02–1.46	0.029	1.09	0.92–1.30	0.332
Age										0.98	0.97–0.99	<0.001	0.97	0.96–0.98	<0.001	0.98	0.97–0.99	0.002
Gender (ref: female)	0.81	0.64–1.04	0.102	0.88	0.70–1.11	0.283	0.72	0.57–0.91	0.005
Marital status (ref: unmarried)									
Married	0.68	0.52–0.89	0.005	0.9	0.69–1.17	0.429	0.81	0.64–1.03	0.089
Divorce or bereaved	1.37	0.87–2.17	0.178	1.34	0.82–2.19	0.243	2.27	1.34–3.85	0.002
Education level (ref: Primary school and less)									
Middle school	0.68	0.54–0.86	0.001	0.96	0.76–1.22	0.726	0.69	0.49–0.97	0.032
High school/vocation school	0.67	0.50–0.89	0.006	0.74	0.55–0.99	0.046	0.74	0.51–1.08	0.124
Junior college	0.61	0.43–0.87	0.006	0.83	0.58–1.19	0.312	0.65	0.42–1.00	0.048
College and more	0.55	0.36–0.84	0.006	0.67	0.43–1.05	0.081	0.64	0.41–0.98	0.042
Labor contract (ref: no)	0.87	0.72–1.04	0.132	1	0.83–1.20	0.999	1.17	0.96–1.43	0.127
Working hours										1.01	1.00–1.01	<0.001	1	1.00–1.01	0.311	1.01	1.00–1.01	0.005
Smoking (ref: no)	1.22	0.94–1.58	0.135	1.2	0.95–1.51	0.133	1.33	1.08–1.63	0.007
Alcohol consumption (ref: no)	0.86	0.63–1.19	0.365	0.94	0.72–1.23	0.652	1.29	1.01–1.63	0.039
Physical exercise (ref: no)										0.76	0.63–0.92	0.006	0.85	0.69–1.05	0.131	1.12	0.93–1.35	0.241
Self-rated health (ref: bad)									
Fair	0.48	0.37–0.64	<0.001	0.45	0.33–0.61	<0.001	0.61	0.45–0.81	0.001
Good	0.24	0.18–0.32	<0.001	0.26	0.19–0.35	<0.001	0.31	0.23–0.42	<0.001
Chronic disease (ref: no)	1.18	0.87–1.60	0.274	1.41	1.00–1.98	0.05	1.75	1.24–2.47	0.001
Self-evaluation income (ref: low)									
Fair	0.74	0.61–0.89	0.001	0.76	0.62–0.93	0.009	0.63	0.50–0.79	<0.001
High	0.68	0.53–0.89	0.004	0.65	0.49–0.86	0.003	0.43	0.33–0.58	<0.001
Occupation type	Control			Control			Control		
Observations	1702			1,663			1,635		

## 5. Discussion

The impact of on-call work on the protection of workers’ labor rights and interests has attracted the attention of the academic community (Chen et al., 2020; Lu and Chen, 2020), but related studies ignore the impact on workers’ mental health. Thus, this study takes rural migrant workers engaged in on-call work as the research object and discusses the impact of this novel and rapidly developing employment model on the mental health of workers. Our research shows that on-call work is closely related to the mental health of rural migrant workers, and on-call work exacerbates the depressive mood of rural migrant workers, a conclusion that still holds after adjusting for related potential factors. On-call work makes employees face more uncertainty than typical work arrangements that have standard work hours. In on-call work, employees may receive work tasks at any time. On the one hand, when they receive work assignments, they have to give up their leisure to increase work hours, which harms work recovery (Yu and Leka, 2022). In recent years, incidents of sudden illness and sudden death from overwork have occurred among gig workers who are on call. On the other hand, even if workers are not called in, the mere possibility of receiving tasks can adversely affect their job recovery, increase stress, and exacerbate depression.

In addition, internet development is closely related to the on-call work model, and this study further conducts a heterogeneity analysis based on the internet usage of rural migrant workers. The results reveal that after adjusting for relevant potential factors, on-call work is more likely to aggravate the depression of rural migrant workers with a high degree of internet use. This is because a large proportion of the on-call workers who are highly dependent on the internet are gig workers. To increase market share and profit growth, the “gig economy” platform reduces the employment threshold (Gussek and Wiesche, 2022) and expands the scale of employment, which intensifies the internal competition among gig workers (Kost et al., 2020) and results in a continuous reduction in earnings (Schwellnus et al., 2019). Most gig work does not guarantee a base salary. Extending working hours and improving labor efficiency are the main ways for gig workers to increase their income, which means they must take on highly intense work and more work than a standard work week. Moreover, there is serious information asymmetry between gig enterprises and on-call workers (Graham and Anwar, 2019), and it is difficult for these workers to form a collective identity (Kost et al., 2020); thus, it is difficult to effectively and clearly strive for reasonable collective interests (Wood et al., 2018; Glavin et al., 2021), which weakens the ability of workers to negotiate with these enterprises. Under such circumstances, on-call workers are faced with greater job competition pressure. The unstable nature of the job (Keith et al., 2020) as well as the low level of bargaining power further deteriorate the psychosocial environment of on-call workers (Demiral et al., 2022). On-call workers with low and medium internet dependence receive work assignments primarily by phone, and they usually receive phone-based assignments less frequently. The on-call tasks performed by these workers are often more traditional routine technical jobs (such as maintenance workers) or additional tasks received outside of standard working hours. The work of these traditional skilled employees requires a certain technical ability, which raises the employment threshold, reduces job competition pressure (Kost et al., 2020), and guarantees their welfare benefits to a certain extent. These effects partially compensate for the negative impact of the uncertainty of on-call work. Furthermore, for rural migrant workers who perform additional tasks outside of standard working hours, the frequency of receiving such work assignments is low. In this case, the impact of 24-h on-call jobs on workers’ emotions is relatively mild. At the same time, through the heterogeneity analysis of the income level of rural migrant workers, on-call work is still an important predisposing factor for depression among rural migrant workers with low and medium wages. However, for rural migrant workers with higher wages, a better income increases their sense of happiness and security, which to a certain extent compensates for the negative impact of on-call work (Thomson et al., 2022); thus, the impact of on-call work on their depression is not obvious. Among rural migrant workers who are closely connected to the internet, on-call work is more likely to cause depression, and the on-call work model is more likely to have a negative impact on the emotional state of low-income workers.

This study focuses on a disadvantaged group of Chinese rural migrant workers. It empirically examines the relationship between on-call work and rural migrant workers’ depression and provides a basis for psychological crisis intervention for Chinese rural migrant workers. However, this study has several limitations. First, in addition to depression, the on-call work model can also cause psychological problems, such as stress, anxiety, and loneliness. Therefore, future research should combine a variety of psychological problems to explore the impact of on-call work on the mental health of workers. Second, the change in depressive mood is a long-term dynamic process. There may be certain limitations in the use of cross-sectional data in this paper. The relevant dynamic analysis needs to be carried out with more comprehensive panel data.

## 6. Conclusion

This study explores the relationship between on-call work and depressive mood among Chinese rural migrant workers and provides evidence of a correlation between the two. The results show that the odds ratio of depressive mood among rural migrant workers who work on call is 1.22 (95% CI 1.04–1.43) compared with rural migrant workers whose work does not require being on call. Furthermore, the study finds that gig work with a strong dependence on the internet is more likely to have a negative impact on the emotions of rural migrant workers, and on-call work is more likely to cause mental health problems in low- and middle-income workers. To alleviate and prevent depression among rural migrant workers, it is necessary to establish a targeted mental health management plan, incorporate depression assessment into routine health examinations, and provide mental health-related services for rural migrant workers with a high depression index. In addition, it is necessary to standardize the employment behavior of gig enterprises, improve the psychosocial work environment of gig workers, give workers appropriate labor compensation for high-frequency on-call tasks, and provide regular relaxation time to guide and help on-call workers achieve work-life balance.

## Data availability statement

Publicly available datasets were analyzed in this study. This data can be found at: https://opendata.pku.edu.cn/dataverse/CFPS.

## Ethics statement

The studies involving human participants were reviewed and approved by Institutional Review Board (or Ethics Committee) of Peking University. Written informed consent to participate in this study was provided by the participants’ legal guardian/next of kin.

## Author contributions

QX and LW: conceptualization and validation. LW: methodology, project administration, and writing—original draft preparation. QX: software, visualization, and funding acquisition. YZ and XJ: formal analysis. YZ: investigation. XJ: resources. LW and YZ: data curation. QX and XJ: writing—review and editing and supervision. All authors contributed to the article and approved the submitted version.

## Funding

This research was funded by the National Social Science Fund of China, grant number 19BSH085.

## Conflict of interest

The authors declare that the research was conducted in the absence of any commercial or financial relationships that could be construed as a potential conflict of interest.

## Publisher’s note

All claims expressed in this article are solely those of the authors and do not necessarily represent those of their affiliated organizations, or those of the publisher, the editors and the reviewers. Any product that may be evaluated in this article, or claim that may be made by its manufacturer, is not guaranteed or endorsed by the publisher.

## Footnotes

^1^On-call work in this article is work that requires workers to be readily available to respond to any arrangement required by the workplace.

^2^In this paper, the term “rural migrant workers” is used to refer to people whose household registration is still in rural areas and who are mainly engaged in nonagricultural industries. Some of them go out to work during the slack farming season, with strong mobility, and some are employed in cities for a long time and come to represent an important part of industrial workers.

## Appendix

Step 1: To eliminate the limitation of the value range of the dependent variable, take

(A1)
$$O_{i}=\frac{P_{i}}{1-P_{i}}$$

In the formula, $P_{i}$ represents the probability of occurrence of the rural migrant worker’s depression degree is i , and $O_{i}$ is the ratio, which refers to the possibility of occurring relative to the possibility of not occurring.

Step 2: Take the logarithm of equation (A1):

(A2)
$$Y_{i}=\ln\left[\frac{P_{i}}{1-P_{i}}\right]$$

At this time, formula (A2) has no upper or lower limits and is symmetrical with 0.5 as the midpoint. A small change in probability will cause a larger change, making the data easier to observe.

Step 3: In the absence of upper and lower limits, the change in Y relative to X can be said to be linearly related.

(A3)
$$\ln\left(\frac{P_{i}}{1-P_{i}}\right)=b_{0}+b_{1}X_{i}=\log itY_{i}$$

where $X_{i}$ is the independent variable, $b_{1}$ is the coefficient of $X_{i}$ , and $b_{0}$ is a constant.

then there are

(A4)
$$P_{i}=\frac{e^{Y_{i}}}{1+e^{Y_{i}}}$$

Equation (A4) is the general expression of the logistic regression model.
